# Adoption of robot-assisted radical nephroureterectomy permits a minimally invasive option for management of upper tract urothelial carcinoma in geriatric patients: comparison with non-geriatric patients with intermediate-term oncologic follow-up

**DOI:** 10.1007/s11701-024-02013-6

**Published:** 2024-06-19

**Authors:** Justin M. Refugia, Parth U. Thakker, Timothy K. O’Rourke, Adam Cohen, Aaron Bradshaw, Randy Casals, Maxwell Sandberg, Wyatt Whitman, Sumit Saini, Ashok K. Hemal

**Affiliations:** Department of Urology, Atrium Health Wake Forest Baptist Medical Center, 140 Charlois Boulevard, Winston-Salem, NC 27103 USA

**Keywords:** Robot-assisted, Nephroureterectomy, Geriatric oncology, Upper tract urothelial carcinoma, Minimally invasive surgery

## Abstract

To assess the oncologic efficacy and safety of robot-assisted approach to radical nephroureterectomy (RARNU) in geriatric versus younger patients with upper tract urothelial carcinoma (UTUC). A single-center, retrospective cohort study was conducted from 2009 to 2022 of 145 patients (two cohorts: < 75 and ≥ 75 years old) with non-metastatic UTUC who underwent RARNU. Primary endpoint was UTUC-related recurrence of disease during surveillance (bladder-specific and metastatic). Safety was assessed according to 30-day, modified Clavien–Dindo (CD) classifications (Major: C.D. III–V). Survival estimates were performed using Kaplan–Meier method. There were 89 patients < 75 years (median 65 years) and 56 patients ≥ 75 years (median 81 years). Comparing the young versus geriatric cohorts: median follow-up 38 vs 24 months (*p* = 0.03, respectively) with similar 3-year bladder-specific recurrence survival (60% vs 67%, HR 0.70, 95% CI [0.35, 1.40], *p* = 0.31) and metastasis-free survival (79% vs 70%, HR 0.71, 95% CI [0.30, 1.70], *p* = 0.44). Expectedly, the younger cohort had a significant deviation in overall survival compared to the geriatric cohort at 1-year (89% vs 76%) and 3-years (72% vs 41%; HR 3.29, 95% CI [1.88, 5.78], *p* < 0.01). The 30-day major (1% vs 0) and minor complications (8% vs 14%, *p* = 0.87). Limitations include retrospective study design of a high-volume, single-surgeon experience. Compared to younger patients with UTUC, geriatric patients undergoing RARNU have similar oncologic outcomes at intermediate-term follow-up with no increased risk of 30-day perioperative complications. Thus, age alone should not be used to disqualify patients from definitive surgical management of UTUC with RARNU.

## Introduction

Choosing to operate on geriatric patients with a malignancy is central to providing inclusive oncologic care. Upper urinary tract urothelial carcinoma (UTUC) is a rare and aggressive urologic malignancy that is increasingly diagnosed in geriatric patients. The incidence of UTUC diagnosis peaks between 70 and 90 years, with a mean age at diagnosis is 73 years [1–3]. Upper urinary tract tumor pathologic staging and grading are the main prognostic factors for cancer-specific survival, independent of age and underlying comorbidities [4, 5]. For non-metastatic UTUC tumors, the standard treatment has been surgical resection with radical nephroureterectomy with bladder cuff excision and regional lymphadenectomy or, in select carefully selected cases, segmental ureterectomy with upper tract reconstruction [6–8]. Robot-assisted laparoscopic radical nephroureterectomy with bladder cuff excision (RARNU) has reduced morbidity, highlighting the effectiveness of minimally invasive surgery as an alternative to traditional open surgery [9].

Advanced age at RARNU is linked to decreased cancer-specific and overall survival, but age along should not be used to exclude patients from the choosing to undergo RARNU [10–13]. A multidimensional geriatric assessment should be considered to determine surgical candidates [14]. RARNU can expand the role of surgical resection for those who may have an increased risk of perioperative complications, such as geriatric patient [15–17]. Furthermore, RARNU has demonstrated similar oncological efficacy to open and laparoscopic approaches for UTUC management [15, 18, 19].

Few studies have examined the effectiveness of RARNU in treating UTUC in geriatric patients compared to younger ones. Our institution routinely offers RARNU for older patients; thus, we aimed to assess if the decision to operate on geriatric patients was associated with worse oncologic outcomes and increased postoperative complications as compared to the younger patients.

## Methods

In this study, we evaluated adult patients with non-metastatic UTUC that underwent RARNU at an academic, tertiary referral hospital. Patient data were extracted from a prospectively maintained database that was designed to capture clinical and pathologic outcomes beginning in January 2009, when RARNU was first performed at our center. Patients required routine staging work up with cystoscopy and cross-sectional imaging to rule out synchronous bladder lesions and/or metastatic disease. Patients with a history of non-muscle invasive bladder cancer (NMIBC) required complete clinical response prior to treatments to be included in the database. This database does not include patients with history of muscle invasive bladder cancer or radical cystectomy.

The study period was between January 2009 and July 2022. All RARNU were performed using a previously described single-docking technique using the different generation of the da Vinci robotic platform (first generation daVinci; daVinci HD; daVinci-SI; daVinci-XI) during the study period (Intuitive Surgical, Inc., Sunnyvale, CA, USA) [20–22]. Bladder cuff excision and closure with absorbable suture was performed in all the included patients. Regional lymphadenectomy was performed at the discretion of the surgeon in cases of patients with bulky and/or T3 disease, high-grade pathology, or radiologic evidence of nodal disease. Perioperative intravesical chemotherapy instillation with gemcitabine or mitomycin C was performed per protocol. Patients with advanced UTUC pathology (pT2–pT4 or any nodal disease) were referred to medical oncology for evaluation to receive neo-adjuvant or adjuvant chemotherapy. Postoperative surveillance was conducted with the first post-operative surveillance visit at 3 months, which included white light cystoscopy, followed by repeat cystoscopy every 6–12 months along with cross-sectional abdominal imaging and chest imaging for upper tract and chest surveillance as per protocol. Patients with recurring bladder tumor underwent resection to confirm a diagnosis of urothelial carcinoma. Metastatic disease presence was identified through radiologist-reviewed surveillance imaging.

### Statistical analysis

The study compared two cohorts of patients based on age (geriatric patients ≥ 75 years, younger patients < 75 years) and primarily analyzed disease recurrence rates. Follow-up was calculated from date of surgery to most recent surveillance evaluation. The cohorts were summarized with descriptive statistics and compared to one another (continuous data: Mann–Whitney *U* test, categorical data: Fisher’s exact test). The Kaplan–Meier method was used to estimate bladder-recurrence-free survival (BRFS), metastasis-free survival (MFS), and disease-free survival (DFS) probabilities at 1- and 3 years post-op. Patients that had no evidence of disease recurrence in the study surveillance period were censored. Log-rank tests were applied to compare the geriatric versus younger cohorts. Non-adjusted hazard ratios were calculated to assess the effect of age on various survival outcomes.

Secondarily, the study evaluated the safety of RARNU and all-cause mortality (Overall Survival, OS). Mortality was defined as the time from surgery to death from any cause, but no data on specific causes were available. Safety was assessed based on Clavien–Dindo complications (Minor: Grades I–II, Major: Grades III–V), blood transfusion rates, and hospital stay lengths. The effect of age on RARNU’s safety was evaluated using univariate statistical testing. All statistical testing was two sided, and significance was marked at *p* < 0.05. Statistical analyses were performed with SAS software (2023, SAS Institute Inc. Cary, NC, USA) and Prism version 8.0.0 (San Diego, California USA).

## Results

**Fig. 1:**
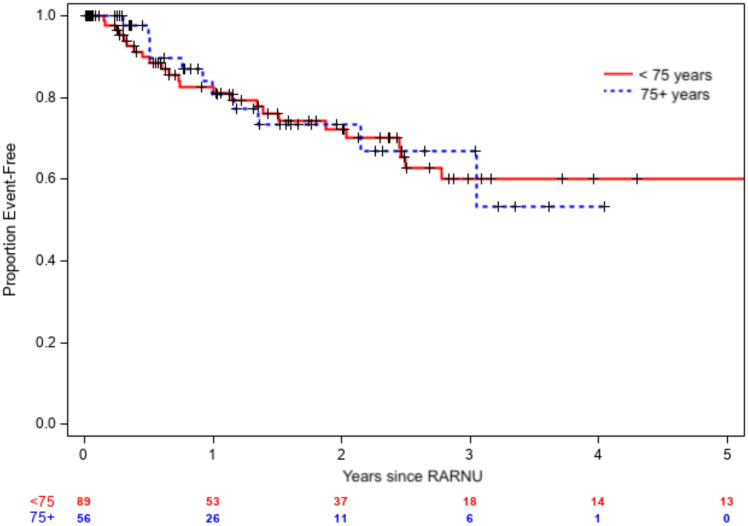
Kaplan–Meier survival estimates for bladder-specific recurrence of disease (BRFS) after RARNU

**Fig. 2:**
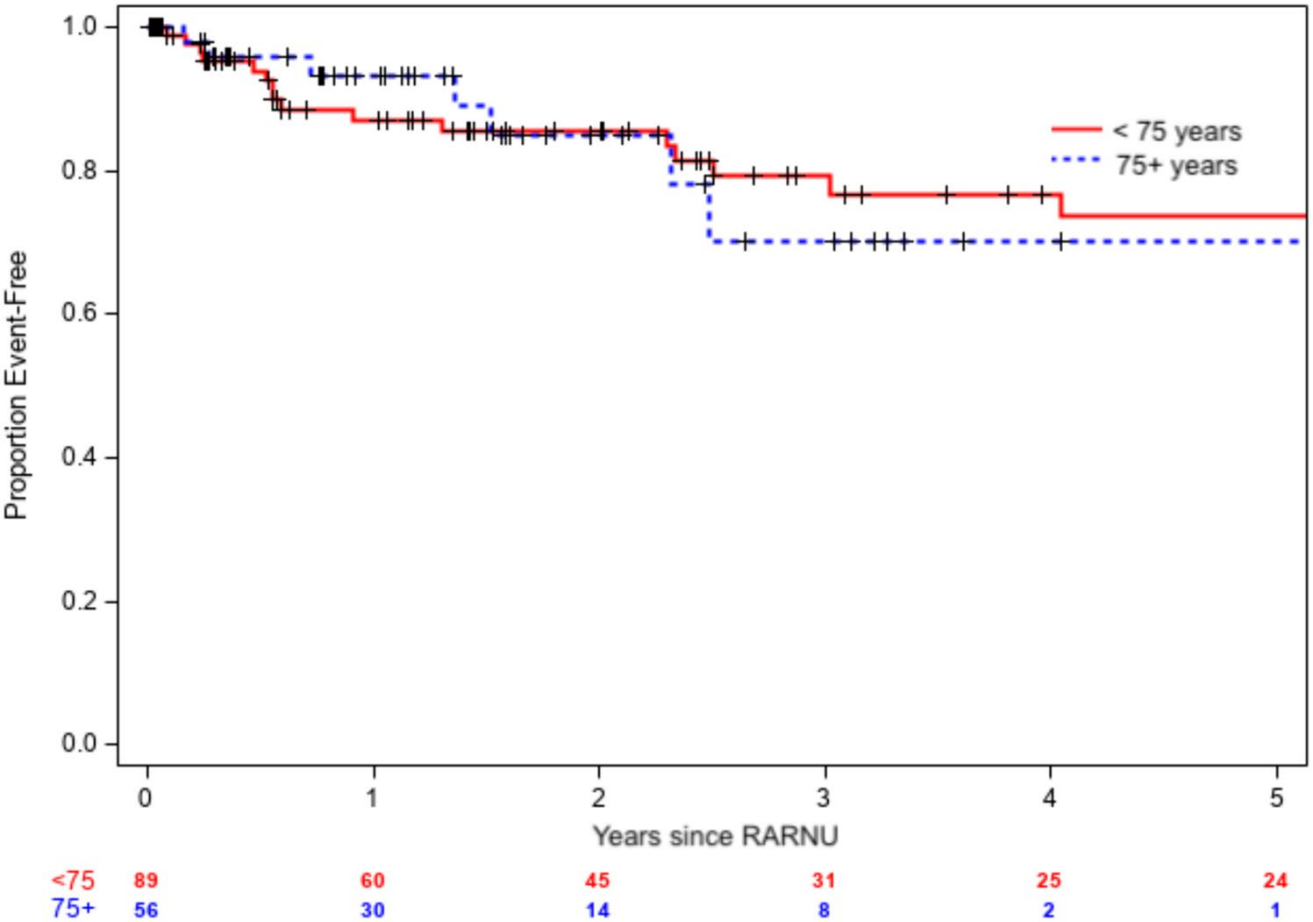
Kaplan–Meier survival estimates for metastasis-specific recurrence of disease (MFS) after RARNU

During the study period, 145 adult patients were included in the database, with 89 in the younger cohort (median age of 65 years) and 56 in the geriatric cohort (median age of 81 years) (Table 1). The primary endpoint of disease recurrence was similar between the two groups. The younger cohort was followed for a median of three years after the day of surgery, during which 36 patients had disease recurrences (25 bladder-specific, 11 metastatic). The geriatric cohort was followed by a median of 2 years and 16 patients had disease recurrences (11 bladder specific, five metastatic). At 1 and 3 years after RARNU, patients in the geriatric cohort had no statistically significant differences in BRFS, MFS, or DFS compared to the younger cohort. (Table 2) For BRFS, 3-year survival estimates were 60% vs 67% (HR 0.70, 95% CI [0.35, 1.40], *p* = 0.3) (Fig. 1). For MFS, 3-year survival estimates were 79% vs 70% (HR 0.71, 95% C.I. [0.30, 1.70], *p* = 0.4). (Fig. 2) The 3-year DFS estimates were 45% vs 51% (HR 0.67, 95% C.I. [0.38, 1.19], *p* = 0.2).

**Table 1:** Baseline clinicopathologic and treatment characteristics of the patients in each cohort (young or geriatric)

Variable	Young *n* = 89	Geriatric *n* = 56	*p*–value
Median age, years	65 (60–71)	81 (78–85)	0.01
Male sex	65 (73)	37 (66)	0.46
Ethnicity			0.90
White	82 (92)	52 (93)	
African American	5 (6)	4 (7)	
Other	2 (2)	0	
Body mass index, kg/m^2^	28 (25–32)	26 (22–29)	0.01
ASA > 3	9 (10)	17 (30)	0.002
Prior NMIBC (treated)	30 (34)	11 (20)	0.07
Pre-RARNU biopsy performed	56 (73)	33 (77)	0.67
Pre-RARNU biopsy pathology			0.65
≤ T1	54 (96)	32 (97)	
T2	1 (2)	1 (3)	
T3	1 (2)	0	
RARNU time, min	185 (141–220)	163 (116–209)	0.08
Ipsilateral lymph node dissection	61 (68)	35 (62)	0.48
Perioperative intravesical chemotherapy	26 (30)	15 (27)	0.96
Length of stays, d	2 (2–3)	2 (2–3)	0.16
Pathologic tumor stage			0.79
≤ T1	45 (50)	29 (52)	
T2	13 (15)	6 (11)	
T3–T4	31 (35)	21 (37)	
High-grade pathology	78 (88)	46 (82)	0.47
Multifocal tumor	17 (19)	8 (14)	0.51
Positive surgical margin	7 (8)	10 (18)	0.11
Node positive	7 (11)	4 (11)	> 0.99
30-day complications			0.87
None	81 (91)	48 (86)	
Minor (Clavien I–II)	7 (8)	8 (14)	
Severe (Clavien III–V)	1 (1)	0	
Received adjuvant chemotherapy	20 (22)	5 (9)	0.04

*ASA* American Society of Anesthesiologists, *RARNU* Robot-assisted radical nephroureterectomy. Continuous variables reported with medians (interquartile range). Categorical variables reported with occurrences (frequency)

**Table 2:** Kaplan–Meier survival estimates following RARNU

Group	Estimated survival rate after RARNU, % (95% CI)		
	1 year	3 years	*P*–value
Bladder-recurrence free			0.31
Non-geriatric	83 (74–91)	60 (47–73)	
Geriatric	81 (68–94)	67 (48–85)	
Metastasis-free			0.44
Non-geriatric	87 (77–94)	79 (69–89)	
Geriatric	93 (86–100)	70 (49–92)	
Disease-free			0.17
Non-geriatric	72 (62–82)	45 (32–58)	
Geriatric	77 (64–90)	51 (31–71)	
Overall			< 0.001
Non-geriatric	89 (82–96)	72 (61–83)	
Geriatric	76 (64–88)	41 (24–58)

In the study, 27 deaths occurred in the younger cohort at a median of 8.6 years, while 28 deaths occurred in the geriatric cohort at a median of 2.3 years. Geriatric age was associated with a significantly lower 3-year all-cause mortality. Geriatric age was not associated with an increased risk of minor or major C.D. complications in the first 30 days after surgery. (Table 1) There were no patient deaths for either cohort in the first 30 days post operatively. Compared to younger patients, those with geriatric age did not have increased rates of perioperative blood transfusions (2% vs 3%, *p* = 0.6) or prolonged hospital lengths of stay (median of two days in both cohorts, *p* = 0.2).

## Discussion

Upper-tract urothelial carcinoma remains a highly aggressive urological malignancy especially in elderly population [3, 5]. The management of non-metastatic, UTUC necessitates counseling based on individual and cancer-specific factors. The standard of care option chosen by many of these patients is radical nephroureterectomy; however, some patients have historically been disqualified from this major operation based on age. Significant disease-related morbidity such as renal obstruction, infected hydronephrosis, flank pain, and hematuria often factor into surgical decision-making for patients with UTUC. Given high acuity of symptomatology these patients are not suitable for neo-adjuvant chemotherapy and require consideration of surgical intervention. With improvement in surgical approaches, RARNU may be an option for older and more frail patients with similar outcomes and rates of complications relative to younger, healthier patients. The perioperative benefits of RARNU compared to open or laparoscopic approaches have been documented and large population-based studies have demonstrated equivalent oncologic outcomes [18, 23]. Our study results suggest that the decision to operate on geriatric patients with UTUC, by means of RARNU, was not associated with increased risk of disease recurrence or 30-day perioperative complications when compared to operating on younger patients with UTUC.

Several study groups have reported on the oncologic outcomes in patients that chose to pursue RARNU, and their results are like those reported in our study. Compared to a subset of 252 patients undergoing RARNU with a median age of 70 years, oncologic outcomes were similar in patients ≥ 75 years in our study [15]. Grossman et al. found a BRFS of 58.9%, RFS of 72.9%, CSS of 85.5%, and OS of 80.9% at three years [15]. Bae et al. found a 3-year BRFS of 70% and OS of 92% in their cohort of 119 patients with a median age of 69 years and Clements et al. found 3-year OS of 67% for 315 patients from a Medicare database [23, 24]. The 3-year BRFS in our geriatric cohort (67%) was comparable or improved compared to those previously reported with lower RFS and OS. An improved BRFS in our geriatric cohort is likely directly related to worse OS. Bladder recurrences occur commonly in patients with UTUC; however, patients with a lower life expectancy may not live long enough to experience a bladder recurrence. Age has been reported as an independent predictor of worse CSS and OS after RNU and the elderly cohort in this study has the highest median age reported thus far in the literature [10–12] Furthermore, upstaging to muscle invasive UTUC in our cohort was 48% in our cohort which is higher than the previously reported 41.7% and may contribute to the lower RFS.

Oncologic outcomes for geriatric patients pursuing RARNU may continue to improve with the implementation of neoadjuvant (NAC), adjuvant chemotherapy (AC), and multidisciplinary evaluation including geriatric evaluation to provide best medical optimization. Evidence from a 2020 systematic review and meta-analysis by Leow et al. detailed the OS and CSS benefit of NAC and AC compared to RNU alone [25]. Furthermore, in a multi-center, retrospective cohort study of elderly (> 68 years) versus non-elderly patients receiving NAC with RNU, there was no difference shown for RFS or CSS, suggesting that older patients may also benefit from NAC [26]. Most patients in our cohort were treated with RARNU prior to the general acceptance of NAC for UTUC, and thus survival estimates may improve with the implementation of NAC.

The decision to offer major surgery to elderly patients has traditionally been thought to warrant counseling on higher rates of post-operative complications than younger patients, though more recent studies have demonstrated equivalent post-operative complication rates [27, 28]. In our study, older patients had a higher median ASA status and lower BMI; however, major and minor perioperative complications rates were not increased relative to the younger patient cohort. Furthermore, our geriatric cohort had a similar frequency of minor and major C.D. complications to those reported in Veccia et al. (14% vs 18%, minor and 0 vs 3%, major) [18]. The frequency of overall 30-day complications in our geriatric cohort was lower than that previously reported. These differences may be attributed to improvement in surgical technique, preoperative optimization, or frailty of geriatric cohort as frailty has been demonstrated to have a strong correlation with post-operative complications [29]. The local protocol at our institution includes pre-operative risk stratification and optimization by both Internal Medicine and Anesthesiology to identify co-morbidities that need to be addressed prior to surgery.

This study has few limitations. First, the study is limited by its retrospective design and the inherent biases associated with it. Next, there is a significant selection bias due to most patients choosing to undergo an operation by our center’s high-volume robotic surgeon that is well versed in RARNU techniques. Thus, results of our study are limited in generalizability particularly to patient outcomes when in the hands of urologists that do not routinely manage UTUC or perform RARNU. Second, our prospectively maintained, nephroureterectomy database has significant heterogeneity regarding the perioperative and postoperative management and surveillance that changed as newer literature emerged, and thus oncologic outcomes may be expected to improve. Finally, our study did not implement a frailty score. Compared to ASA status, frailty scores may better predict perioperative complications and as such, future studies may benefit from the inclusion of comorbidity indices.

Nonetheless, this study includes a patient cohort with a higher median age than any previously reported, to the best of our knowledge. Understanding the role of surgery in patients with malignancy, such as UTUC, is critical due to growing prevalence of UTUC in the aging population. Our study highlights that the choice to perform surgery in this case with RARNU on patients ≥ 75 years with non-metastatic UTUC was not associated with increased risks of oncologic disease recurrence or post-operative complications. The differences in OS were in line with prior studies, thus validating the importance in counseling patients of advanced age regarding preoperative optimization and proper patient selection [14]. Advances in surgical techniques for RARNU, such as complete retroperitoneal access and use of single-port system, as well as the use of NAC, may improve oncologic parameters and decrease perioperative complications in this age group; however, further studies are needed [16].

## Conclusions

At intermediate-term oncologic follow-up, our data suggest that the advanced age of geriatric patients choosing to undergo RARNU for non-metastatic, UTUC was not associated with higher risk of worse oncologic outcomes nor associated with increased risk of perioperative complications. Future clinical studies are warranted to determine factors that increase the risk of UTUC-related recurrences in geriatric patients at long-term oncologic follow-up.

## Data Availability

No datasets were generated or analysed during the current study.
